# 2-[2-(1*H*-Imidazol-3-ium-5-yl)eth­yl]-3-(pyridin-2-yl)-2*H*-imidazo[1,5-*a*]pyridin-4-ium bis­(perchlorate)

**DOI:** 10.1107/S1600536811015625

**Published:** 2011-04-29

**Authors:** Murat Türkyılmaz, Yakup Baran, Namık Özdemir

**Affiliations:** a Department of Chemistry, Faculty of Science, Trakya University, 22030 Edirne, Turkey; b Department of Chemistry, Faculty of Arts and Sciences, Çanakkale Onsekiz Mart University, 17020 Çanakkale, Turkey; c Department of Physics, Faculty of Arts and Sciences, Ondokuz Mayıs University, 55139 Samsun, Turkey

## Abstract

In the title mol­ecular salt, C_17_H_17_N_5_^+^·2ClO_4_^−^, the dihedral angles between the fused-ring system and the pendant five- and six-membered heterocyclic rings are 6.4 (2) and 41.29 (19)°, respectively. The O atoms of both perchlorate anions are disordered over two sets of sites with occupancy ratios of 0.614 (8):0.386 (8) and 0.591 (7):0.409 (7). An intra­molecular C—H⋯N contact occurs in the cation. In the crystal, the components are linked by N—H⋯O and C—H⋯O hydrogen bonds and π–π stacking inter­actions [centroid–centroid separation = 3.642 (3) Å].

## Related literature

For background to the biological properties of imidazopyridine compounds, see: Kaminski & Doweyko (1997 ▶); Sanfillipo *et al.* (1988 ▶); Lhassani *et al.* (1999 ▶). For hydrogen-bond motifs, see: Bernstein *et al.* (1995 ▶).
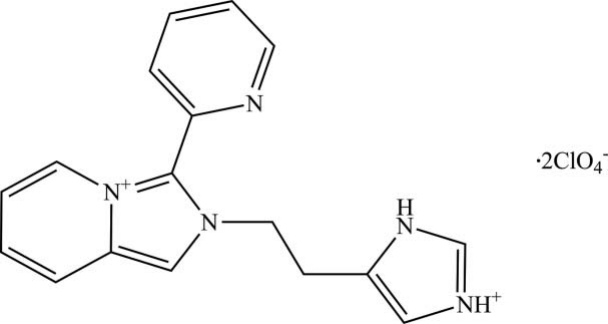


## Experimental

### 

#### Crystal data

C_17_H_17_N_5_^2+^·2ClO_4_^−^
*M* *_r_* = 490.26
Monoclinic, 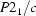

*a* = 15.044 (6) Å
*b* = 11.303 (4) Å
*c* = 12.783 (5) Å
β = 108.009 (15)°
*V* = 2067.1 (13) Å^3^
*Z* = 4
Mo *K*α radiation
μ = 0.37 mm^−1^
*T* = 273 K
0.35 × 0.20 × 0.20 mm

#### Data collection

Rigaku R-AXIS RAPID diffractometer
19199 measured reflections
4507 independent reflections
2540 reflections with *I* > 2σ(*I*)
*R*_int_ = 0.083

#### Refinement

*R*[*F* ^2^ > 2σ(*F* ^2^)] = 0.074
*wR*(*F* ^2^) = 0.214
*S* = 1.07
4507 reflections
363 parameters
198 restraints
H-atom parameters constrained
Δρ_max_ = 0.45 e Å^−3^
Δρ_min_ = −0.52 e Å^−3^

### 

Data collection: *PROCESS-AUTO* (Rigaku, 1998 ▶); cell refinement: *PROCESS-AUTO*; data reduction: *CrystalStructure* (Rigaku/MSC, 2004) ▶; program(s) used to solve structure: *SHELXS97* (Sheldrick, 2008 ▶); program(s) used to refine structure: *SHELXL97* (Sheldrick, 2008 ▶); molecular graphics: *ORTEP-3* (Farrugia, 1997 ▶); software used to prepare material for publication: *WinGX* (Farrugia, 1999 ▶) and *PLATON* (Spek, 2009 ▶).

## Supplementary Material

Crystal structure: contains datablocks I, global. DOI: 10.1107/S1600536811015625/hb5862sup1.cif

Structure factors: contains datablocks I. DOI: 10.1107/S1600536811015625/hb5862Isup2.hkl

Supplementary material file. DOI: 10.1107/S1600536811015625/hb5862Isup3.cml

Additional supplementary materials:  crystallographic information; 3D view; checkCIF report

## Figures and Tables

**Table 1:** Hydrogen-bond geometry (Å, °)

*D*—H⋯*A*	*D*—H	H⋯*A*	*D*⋯*A*	*D*—H⋯*A*
C13—H13*A*⋯N3	0.97	2.48	3.001 (6)	113
C16—H16⋯O1*A*	0.93	2.44	3.351 (8)	167
N4—H4*N*⋯O7*A*	0.86	1.97	2.807 (10)	165
N5—H5*N*⋯O4*A*^i^	0.86	2.18	2.927 (16)	145
C11—H11⋯O6*A*^ii^	0.93	2.46	3.224 (9)	139
C6—H6⋯O5*A*^iii^	0.93	2.60	3.331 (17)	136
C5—H5⋯O6*A*^iv^	0.93	2.43	3.139 (8)	133

Symmetry codes: (i) 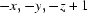; (ii) 
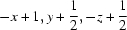
; (iii) 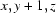; (iv) 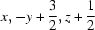.

**Fig. 1.**
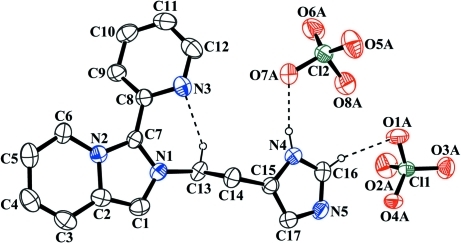
The molecular structure of (I) showing 30% probability displacement ellipsoids. For the sake of clarity, only H atoms involved in hydrogen bonding have been included and only the major parts of disordered fragments are drawn. Hydrogen bonds are indicated by broken lines.

## supplementary crystallographic information

### Comment

Imidazopyridine derivatives are of great importance because of their remarkable biological properties. For example, gastric antisecretory (Kaminski & Doweyko, 1997), local anesthetic (Sanfillipo *et al.*, 1988) and antiviral (Lhassani *et al.*, 1999) properties of imidazo[1,2-*a*]pyridine derivatives have been described.

The title salt comprises a double protonated 2-[2-(1*H*-imidazol-3-ium-5-yl)ethyl]-3-(pyridin-2-yl)-2*H*-imidazo [1,5-*a*]pyridin-4-ium cation and two perchlorate anion (Fig. 1). The interatomic distances and angles in the title salt show no anomalies.

The molecular structure of the title compound, (I), contains one intramolecular C—H···N contact leading to the formation of a six-membered ring with graph-set descriptor S(6) (Bernstein *et al.*, 1995). In the crystal structure, intermolecular C—H···O and N—H···O type hydrogen bonds and π—π stacking interactions between the (N2/C2—C6) and (N3/C8—C12) pyridine rings interconnect the ions into a three-dimensional supramolecular structure (Table 1).

### Experimental

Histamine-HCl (8 mmol, 1.18 g) was dissolved in argon saturated methanol and the solution was placed on a magnetic stirrer at room temperature. 2-Pyridinecarboxaldehyde (10 mmol, 1.08 g) was dissolved in argon saturated methanol and this solution was added to the histamine solution slowly. The final solution was left on a magnetic stirrer and temperature was raised to 333 K and the solution was left there for 24 h. Solvent volume was reduced and the solution was left for crystallization. After several days, brown blocks of (I) were separated, which were collected and dried.

### Refinement

H atoms were positioned geometrically and treated using a riding model, fixing the bond lengths at 0.86, 0.93 and 0.97 Å for NH, CH and CH_2_ groups, respectively. The displacement parameters of the H atoms were constrained as *U*_iso_(H)= 1.2*U*_eq_. In the compound, the O atoms of the two perchlorate anions show positional disorder and the refined site-occupancy factors of the disordered parts, *viz*. (O1A—O4A/O1B—O4B) and (O5A—O8A/O5B—O8B), are 0.614 (8)/0.386 (8)% and 0.591 (7)/0.409 (7)%, respectively.

### Crystal data

**Table:**

C_17_H_17_N_5_^2+^·2ClO_4_^−^	*F*(000) = 1008
*M**_r_* = 490.26	*D*_x_ = 1.575 Mg m^−^^3^
Monoclinic, *P*2_1_/*c*	Mo *K*α radiation, λ = 0.71073 Å
Hall symbol: -P 2ybc	Cell parameters from 11579 reflections
*a* = 15.044 (6) Å	θ = 3.1–29.6°
*b* = 11.303 (4) Å	µ = 0.37 mm^−^^1^
*c* = 12.783 (5) Å	*T* = 273 K
β = 108.009 (15)°	Block, brown
*V* = 2067.1 (13) Å^3^	0.35 × 0.20 × 0.20 mm
*Z* = 4	

### Data collection

**Table:**

Rigaku R-AXIS RAPID diffractometer	2540 reflections with *I* > 2σ(*I*)
Radiation source: fine-focus sealed tube	*R*_int_ = 0.083
graphite	θ_max_ = 27.0°, θ_min_ = 3.1°
Detector resolution: 10.00 pixels mm^-1^	*h* = −19→19
ω scans	*k* = −14→14
19199 measured reflections	*l* = −16→16
4507 independent reflections	

### Refinement

**Table:**

Refinement on *F*^2^	Primary atom site location: structure-invariant direct methods
Least-squares matrix: full	Secondary atom site location: difference Fourier map
*R*[*F*^2^ > 2σ(*F*^2^)] = 0.074	Hydrogen site location: inferred from neighbouring sites
*wR*(*F*^2^) = 0.214	H-atom parameters constrained
*S* = 1.07	*w* = 1/[σ^2^(*F*_o_^2^) + (0.0979*P*)^2^ + 0.8991*P*] where *P* = (*F*_o_^2^ + 2*F*_c_^2^)/3
4507 reflections	(Δ/σ)_max_ < 0.001
363 parameters	Δρ_max_ = 0.45 e Å^−^^3^
198 restraints	Δρ_min_ = −0.52 e Å^−^^3^

### Special details

**Table:**

Geometry. All e.s.d.'s (except the e.s.d. in the dihedral angle between two l.s. planes) are estimated using the full covariance matrix. The cell e.s.d.'s are taken into account individually in the estimation of e.s.d.'s in distances, angles and torsion angles; correlations between e.s.d.'s in cell parameters are only used when they are defined by crystal symmetry. An approximate (isotropic) treatment of cell e.s.d.'s is used for estimating e.s.d.'s involving l.s. planes.
Refinement. Refinement of *F*^2^ against ALL reflections. The weighted *R*-factor *wR* and goodness of fit *S* are based on *F*^2^, conventional *R*-factors *R* are based on *F*, with *F* set to zero for negative *F*^2^. The threshold expression of *F*^2^ > σ(*F*^2^) is used only for calculating *R*-factors(gt) *etc*. and is not relevant to the choice of reflections for refinement. *R*-factors based on *F*^2^ are statistically about twice as large as those based on *F*, and *R*- factors based on ALL data will be even larger.

### Fractional atomic coordinates and isotropic or equivalent isotropic displacement parameters (Å^2^)

**Table:**

	*x*	*y*	*z*	*U*_iso_*/*U*_eq_	Occ. (<1)
Cl1	0.04647 (8)	−0.10009 (10)	0.30270 (8)	0.0598 (4)	
Cl2	0.38314 (10)	0.18832 (12)	0.36810 (11)	0.0791 (4)	
O1A	0.1400 (4)	−0.0618 (7)	0.3141 (7)	0.0894 (19)	0.614 (8)
O2A	−0.0092 (7)	−0.0565 (8)	0.2027 (5)	0.115 (2)	0.614 (8)
O3A	0.0469 (7)	−0.2260 (4)	0.3022 (9)	0.091 (2)	0.614 (8)
O4A	0.0210 (9)	−0.0453 (14)	0.3907 (6)	0.069 (2)	0.614 (8)
O1B	0.0926 (10)	−0.0210 (9)	0.2511 (10)	0.096 (2)	0.386 (8)
O2B	−0.0448 (5)	−0.1148 (12)	0.2321 (8)	0.088 (3)	0.386 (8)
O3B	0.0868 (11)	−0.2140 (7)	0.3301 (15)	0.092 (3)	0.386 (8)
O4B	0.0434 (15)	−0.064 (2)	0.4091 (8)	0.064 (3)	0.386 (8)
O5A	0.4484 (9)	0.1176 (9)	0.4445 (8)	0.114 (3)	0.591 (7)
O6A	0.3768 (6)	0.2058 (7)	0.2528 (5)	0.113 (2)	0.591 (7)
O7A	0.3632 (7)	0.2974 (6)	0.4153 (7)	0.089 (2)	0.591 (7)
O8A	0.2969 (5)	0.1162 (7)	0.3466 (6)	0.0921 (19)	0.591 (7)
O5B	0.4416 (12)	0.0902 (12)	0.4024 (12)	0.105 (3)	0.409 (7)
O6B	0.4497 (7)	0.2597 (8)	0.3319 (10)	0.108 (3)	0.409 (7)
O7B	0.3793 (11)	0.2623 (10)	0.4594 (8)	0.089 (3)	0.409 (7)
O8B	0.2861 (6)	0.1717 (13)	0.3009 (12)	0.123 (3)	0.409 (7)
N1	0.2084 (2)	0.6878 (3)	0.5373 (3)	0.0568 (9)	
N2	0.2744 (2)	0.8592 (3)	0.5682 (3)	0.0525 (8)	
N3	0.3643 (3)	0.6138 (3)	0.4591 (3)	0.0678 (10)	
N4	0.1981 (3)	0.2840 (3)	0.4732 (3)	0.0602 (9)	
H4N	0.2431	0.2966	0.4465	0.072*	
N5	0.0837 (3)	0.2016 (3)	0.5074 (3)	0.0703 (11)	
H5N	0.0406	0.1515	0.5070	0.084*	
C1	0.1678 (3)	0.7453 (4)	0.6032 (3)	0.0633 (12)	
H1	0.1215	0.7153	0.6299	0.076*	
C2	0.2065 (3)	0.8542 (4)	0.6235 (3)	0.0586 (11)	
C3	0.1957 (4)	0.9540 (5)	0.6846 (4)	0.0705 (13)	
H3	0.1515	0.9533	0.7218	0.085*	
C4	0.2487 (4)	1.0493 (5)	0.6890 (4)	0.0786 (15)	
H4	0.2398	1.1161	0.7270	0.094*	
C5	0.3187 (4)	1.0491 (4)	0.6361 (4)	0.0753 (14)	
H5	0.3571	1.1151	0.6429	0.090*	
C6	0.3313 (3)	0.9565 (4)	0.5763 (3)	0.0616 (11)	
H6	0.3771	0.9580	0.5414	0.074*	
C7	0.2737 (3)	0.7553 (3)	0.5146 (3)	0.0519 (10)	
C8	0.3311 (3)	0.7255 (4)	0.4459 (3)	0.0527 (10)	
C9	0.3495 (3)	0.8053 (4)	0.3742 (4)	0.0624 (11)	
H9	0.3223	0.8800	0.3651	0.075*	
C10	0.4094 (4)	0.7724 (5)	0.3157 (4)	0.0763 (14)	
H10	0.4256	0.8254	0.2691	0.092*	
C11	0.4437 (4)	0.6597 (6)	0.3288 (5)	0.0821 (16)	
H11	0.4839	0.6347	0.2907	0.099*	
C12	0.4184 (4)	0.5831 (5)	0.3986 (4)	0.0814 (15)	
H12	0.4404	0.5057	0.4037	0.098*	
C13	0.1788 (3)	0.5677 (4)	0.4936 (4)	0.0613 (11)	
H13A	0.2000	0.5529	0.4304	0.074*	
H13B	0.1112	0.5629	0.4697	0.074*	
C14	0.2189 (3)	0.4744 (4)	0.5806 (4)	0.0643 (12)	
H14A	0.2856	0.4673	0.5932	0.077*	
H14B	0.2091	0.4979	0.6492	0.077*	
C15	0.1729 (3)	0.3587 (4)	0.5444 (3)	0.0554 (10)	
C16	0.1433 (3)	0.1910 (4)	0.4521 (4)	0.0674 (12)	
H16	0.1463	0.1285	0.4060	0.081*	
C17	0.1007 (3)	0.3047 (4)	0.5659 (4)	0.0660 (12)	
H17	0.0683	0.3325	0.6122	0.079*	

### Atomic displacement parameters (Å^2^)

**Table:**

	*U*^11^	*U*^22^	*U*^33^	*U*^12^	*U*^13^	*U*^23^
Cl1	0.0669 (7)	0.0630 (7)	0.0559 (6)	0.0019 (5)	0.0283 (5)	−0.0003 (5)
Cl2	0.0943 (9)	0.0704 (8)	0.0883 (9)	0.0208 (7)	0.0509 (8)	0.0218 (6)
O1A	0.080 (3)	0.106 (4)	0.101 (4)	−0.012 (3)	0.056 (3)	−0.012 (4)
O2A	0.137 (5)	0.124 (5)	0.068 (3)	0.020 (4)	0.006 (3)	0.017 (3)
O3A	0.116 (6)	0.064 (3)	0.094 (5)	−0.010 (3)	0.036 (5)	−0.007 (3)
O4A	0.072 (6)	0.074 (5)	0.072 (3)	−0.001 (4)	0.040 (4)	−0.005 (4)
O1B	0.122 (5)	0.102 (5)	0.083 (5)	−0.009 (4)	0.059 (4)	0.011 (4)
O2B	0.090 (4)	0.108 (6)	0.057 (4)	0.004 (4)	0.010 (3)	−0.016 (4)
O3B	0.107 (7)	0.069 (4)	0.090 (6)	0.020 (4)	0.014 (6)	−0.004 (4)
O4B	0.071 (7)	0.072 (6)	0.050 (3)	−0.007 (5)	0.023 (4)	−0.003 (4)
O5A	0.106 (4)	0.092 (5)	0.122 (6)	0.031 (4)	0.005 (5)	0.007 (4)
O6A	0.149 (5)	0.109 (5)	0.102 (3)	0.040 (4)	0.071 (4)	0.024 (3)
O7A	0.098 (5)	0.061 (3)	0.117 (5)	0.006 (3)	0.047 (5)	0.009 (3)
O8A	0.098 (3)	0.090 (4)	0.084 (4)	0.007 (3)	0.022 (3)	−0.004 (3)
O5B	0.111 (5)	0.082 (5)	0.124 (7)	0.034 (5)	0.038 (6)	0.013 (5)
O6B	0.139 (5)	0.098 (5)	0.118 (6)	0.011 (4)	0.087 (4)	0.020 (5)
O7B	0.091 (5)	0.083 (6)	0.106 (5)	−0.001 (5)	0.050 (5)	−0.003 (4)
O8B	0.117 (4)	0.115 (7)	0.114 (6)	0.018 (4)	0.002 (5)	−0.001 (5)
N1	0.060 (2)	0.056 (2)	0.054 (2)	−0.0036 (17)	0.0177 (18)	0.0058 (16)
N2	0.061 (2)	0.052 (2)	0.0438 (18)	0.0022 (16)	0.0154 (16)	0.0031 (15)
N3	0.073 (3)	0.061 (2)	0.066 (2)	0.0037 (19)	0.017 (2)	−0.0068 (19)
N4	0.065 (2)	0.058 (2)	0.062 (2)	−0.0012 (18)	0.0260 (19)	0.0045 (18)
N5	0.060 (2)	0.057 (2)	0.094 (3)	−0.0071 (18)	0.024 (2)	0.015 (2)
C1	0.062 (3)	0.077 (3)	0.051 (2)	0.002 (2)	0.019 (2)	0.011 (2)
C2	0.058 (3)	0.073 (3)	0.043 (2)	0.012 (2)	0.013 (2)	0.011 (2)
C3	0.077 (3)	0.086 (4)	0.049 (2)	0.019 (3)	0.019 (2)	0.000 (2)
C4	0.099 (4)	0.071 (3)	0.060 (3)	0.018 (3)	0.017 (3)	−0.008 (2)
C5	0.098 (4)	0.054 (3)	0.063 (3)	0.000 (3)	0.009 (3)	−0.005 (2)
C6	0.078 (3)	0.050 (2)	0.054 (2)	−0.007 (2)	0.017 (2)	0.000 (2)
C7	0.057 (2)	0.049 (2)	0.048 (2)	−0.0030 (19)	0.0136 (19)	0.0069 (18)
C8	0.054 (2)	0.053 (2)	0.049 (2)	−0.0041 (19)	0.0132 (19)	−0.0070 (19)
C9	0.065 (3)	0.071 (3)	0.053 (2)	−0.007 (2)	0.022 (2)	−0.008 (2)
C10	0.077 (3)	0.092 (4)	0.063 (3)	−0.027 (3)	0.025 (3)	−0.010 (3)
C11	0.063 (3)	0.105 (5)	0.085 (4)	−0.014 (3)	0.033 (3)	−0.031 (3)
C12	0.071 (3)	0.084 (4)	0.086 (4)	0.007 (3)	0.020 (3)	−0.026 (3)
C13	0.067 (3)	0.055 (3)	0.059 (3)	−0.014 (2)	0.014 (2)	0.007 (2)
C14	0.074 (3)	0.056 (3)	0.061 (3)	−0.003 (2)	0.017 (2)	0.007 (2)
C15	0.058 (3)	0.056 (2)	0.054 (2)	0.000 (2)	0.020 (2)	0.006 (2)
C16	0.072 (3)	0.053 (3)	0.073 (3)	−0.002 (2)	0.017 (3)	0.001 (2)
C17	0.066 (3)	0.065 (3)	0.075 (3)	0.004 (2)	0.034 (3)	0.008 (2)

### Geometric parameters (Å, °)

**Table:**

Cl1—O2A	1.385 (5)	N5—H5N	0.8600
Cl1—O2B	1.401 (6)	C1—C2	1.352 (6)
Cl1—O1B	1.414 (6)	C1—H1	0.9300
Cl1—O3B	1.420 (6)	C2—C3	1.410 (6)
Cl1—O3A	1.423 (5)	C3—C4	1.330 (7)
Cl1—O4A	1.436 (4)	C3—H3	0.9300
Cl1—O4B	1.436 (5)	C4—C5	1.417 (7)
Cl1—O1A	1.435 (5)	C4—H4	0.9300
Cl2—O5B	1.400 (6)	C5—C6	1.343 (6)
Cl2—O5A	1.401 (5)	C5—H5	0.9300
Cl2—O7A	1.444 (5)	C6—H6	0.9300
Cl2—O7B	1.452 (6)	C7—C8	1.448 (5)
Cl2—O8B	1.459 (7)	C8—C9	1.374 (6)
Cl2—O6A	1.461 (5)	C9—C10	1.388 (6)
Cl2—O6B	1.468 (6)	C9—H9	0.9300
Cl2—O8A	1.484 (6)	C10—C11	1.365 (8)
N1—C7	1.344 (5)	C10—H10	0.9300
N1—C1	1.350 (5)	C11—C12	1.379 (8)
N1—C13	1.483 (5)	C11—H11	0.9300
N2—C7	1.358 (5)	C12—H12	0.9300
N2—C6	1.377 (5)	C13—C14	1.515 (6)
N2—C2	1.411 (5)	C13—H13A	0.9700
N3—C12	1.331 (6)	C13—H13B	0.9700
N3—C8	1.349 (5)	C14—C15	1.486 (6)
N4—C16	1.312 (6)	C14—H14A	0.9700
N4—C15	1.377 (5)	C14—H14B	0.9700
N4—H4N	0.8600	C15—C17	1.347 (6)
N5—C16	1.307 (6)	C16—H16	0.9300
N5—C17	1.366 (6)	C17—H17	0.9300
			
O2B—Cl1—O1B	107.4 (7)	C3—C4—H4	119.9
O2B—Cl1—O3B	108.1 (7)	C5—C4—H4	119.9
O1B—Cl1—O3B	117.0 (9)	C6—C5—C4	121.9 (5)
O2A—Cl1—O3A	110.7 (5)	C6—C5—H5	119.1
O2A—Cl1—O4A	109.8 (6)	C4—C5—H5	119.1
O3A—Cl1—O4A	116.0 (8)	C5—C6—N2	118.2 (4)
O2B—Cl1—O4B	109.4 (9)	C5—C6—H6	120.9
O1B—Cl1—O4B	115.2 (12)	N2—C6—H6	120.9
O3B—Cl1—O4B	99.4 (12)	N1—C7—N2	105.9 (4)
O2A—Cl1—O1A	106.3 (5)	N1—C7—C8	127.7 (4)
O3A—Cl1—O1A	107.2 (5)	N2—C7—C8	126.4 (4)
O4A—Cl1—O1A	106.3 (7)	N3—C8—C9	123.7 (4)
O5A—Cl2—O7A	112.8 (5)	N3—C8—C7	114.1 (4)
O5B—Cl2—O7B	112.5 (8)	C9—C8—C7	122.1 (4)
O5B—Cl2—O8B	120.0 (10)	C8—C9—C10	118.8 (5)
O7B—Cl2—O8B	105.4 (9)	C8—C9—H9	120.6
O5A—Cl2—O6A	126.4 (6)	C10—C9—H9	120.6
O7A—Cl2—O6A	110.4 (4)	C11—C10—C9	117.9 (5)
O5B—Cl2—O6B	96.3 (10)	C11—C10—H10	121.0
O7B—Cl2—O6B	97.9 (7)	C9—C10—H10	121.0
O8B—Cl2—O6B	122.8 (8)	C10—C11—C12	119.7 (5)
O5A—Cl2—O8A	101.5 (7)	C10—C11—H11	120.2
O7A—Cl2—O8A	105.3 (5)	C12—C11—H11	120.2
O6A—Cl2—O8A	96.1 (5)	N3—C12—C11	123.6 (5)
C7—N1—C1	111.3 (4)	N3—C12—H12	118.2
C7—N1—C13	126.3 (4)	C11—C12—H12	118.2
C1—N1—C13	122.3 (4)	N1—C13—C14	110.9 (3)
C7—N2—C6	129.5 (4)	N1—C13—H13A	109.5
C7—N2—C2	108.9 (4)	C14—C13—H13A	109.5
C6—N2—C2	121.5 (4)	N1—C13—H13B	109.5
C12—N3—C8	116.1 (4)	C14—C13—H13B	109.5
C16—N4—C15	110.1 (4)	H13A—C13—H13B	108.1
C16—N4—H4N	125.0	C15—C14—C13	110.1 (4)
C15—N4—H4N	125.0	C15—C14—H14A	109.6
C16—N5—C17	109.3 (4)	C13—C14—H14A	109.6
C16—N5—H5N	125.3	C15—C14—H14B	109.6
C17—N5—H5N	125.3	C13—C14—H14B	109.6
N1—C1—C2	107.9 (4)	H14A—C14—H14B	108.2
N1—C1—H1	126.1	C17—C15—N4	105.1 (4)
C2—C1—H1	126.1	C17—C15—C14	131.5 (4)
C1—C2—C3	135.9 (5)	N4—C15—C14	123.3 (4)
C1—C2—N2	106.0 (4)	N5—C16—N4	107.9 (4)
C3—C2—N2	118.1 (4)	N5—C16—H16	126.0
C4—C3—C2	120.1 (5)	N4—C16—H16	126.0
C4—C3—H3	120.0	C15—C17—N5	107.5 (4)
C2—C3—H3	120.0	C15—C17—H17	126.2
C3—C4—C5	120.2 (5)	N5—C17—H17	126.2
			
C7—N1—C1—C2	0.8 (5)	C12—N3—C8—C7	−179.2 (4)
C13—N1—C1—C2	−176.6 (4)	N1—C7—C8—N3	−41.5 (6)
N1—C1—C2—C3	−179.7 (5)	N2—C7—C8—N3	139.5 (4)
N1—C1—C2—N2	−1.2 (4)	N1—C7—C8—C9	138.6 (4)
C7—N2—C2—C1	1.3 (4)	N2—C7—C8—C9	−40.4 (6)
C6—N2—C2—C1	−176.5 (4)	N3—C8—C9—C10	−3.5 (7)
C7—N2—C2—C3	−179.9 (4)	C7—C8—C9—C10	176.4 (4)
C6—N2—C2—C3	2.4 (6)	C8—C9—C10—C11	3.0 (7)
C1—C2—C3—C4	178.3 (5)	C9—C10—C11—C12	−0.1 (7)
N2—C2—C3—C4	−0.1 (6)	C8—N3—C12—C11	2.4 (7)
C2—C3—C4—C5	−2.5 (7)	C10—C11—C12—N3	−2.8 (8)
C3—C4—C5—C6	3.0 (8)	C7—N1—C13—C14	104.7 (5)
C4—C5—C6—N2	−0.7 (7)	C1—N1—C13—C14	−78.3 (5)
C7—N2—C6—C5	−179.2 (4)	N1—C13—C14—C15	167.9 (4)
C2—N2—C6—C5	−2.0 (6)	C16—N4—C15—C17	0.7 (5)
C1—N1—C7—N2	0.1 (5)	C16—N4—C15—C14	−177.3 (4)
C13—N1—C7—N2	177.3 (3)	C13—C14—C15—C17	−96.1 (6)
C1—N1—C7—C8	−179.1 (4)	C13—C14—C15—N4	81.3 (5)
C13—N1—C7—C8	−1.9 (7)	C17—N5—C16—N4	0.0 (5)
C6—N2—C7—N1	176.7 (4)	C15—N4—C16—N5	−0.4 (5)
C2—N2—C7—N1	−0.8 (4)	N4—C15—C17—N5	−0.7 (5)
C6—N2—C7—C8	−4.1 (7)	C14—C15—C17—N5	177.1 (4)
C2—N2—C7—C8	178.4 (4)	C16—N5—C17—C15	0.5 (5)
C12—N3—C8—C9	0.8 (6)		

### Hydrogen-bond geometry (Å, °)

**Table:**

*D*—H···*A*	*D*—H	H···*A*	*D*···*A*	*D*—H···*A*
C13—H13A···N3	0.97	2.48	3.001 (6)	113
C16—H16···O1A	0.93	2.44	3.351 (8)	167
N4—H4N···O7A	0.86	1.97	2.807 (10)	165
N5—H5N···O4A^i^	0.86	2.18	2.927 (16)	145
C11—H11···O6A^ii^	0.93	2.46	3.224 (9)	139
C6—H6···O5A^iii^	0.93	2.60	3.331 (17)	136
C5—H5···O6A^iv^	0.93	2.43	3.139 (8)	133

Symmetry codes: (i) −*x*, −*y*, −*z*+1; (ii) −*x*+1, *y*+1/2, −*z*+1/2; (iii) *x*, *y*+1, *z*; (iv) *x*, −*y*+3/2, *z*+1/2.

## References

[bb1] Bernstein, J., Davis, R. E., Shimoni, L. & Chang, N. L. (1995). *Angew. Chem. Int. Ed. Engl.* **34**, 1555–1573.

[bb2] Farrugia, L. J. (1997). *J. Appl. Cryst.* **30**, 565.

[bb3] Farrugia, L. J. (1999). *J. Appl. Cryst.* **32**, 837–838.

[bb4] Kaminski, J. J. & Doweyko, A. M. (1997). *J. Med. Chem.* **40**, 427–436. 10.1021/jm950700s 9046332

[bb5] Lhassani, M., Chavignon, O., Chezal, J.-M., Teulade, J.-C., Chapat, J.-P., Snoeck, R., Andrei, G., Balzarini, J., De Clercq, E. & Gueiffier, A. (1999). *Eur. J. Med. Chem.* **34**, 271–274. 10.1021/jm981051y 9836626

[bb6] Rigaku (1998). *PROCESS-AUTO* Rigaku Corporation, Tokyo, Japan.

[bb7] Rigaku/MSC (2004). *CrystalStructure* Rigaku/MSC, The Woodlands, Texas, USA.

[bb8] Sanfillipo, P. J., Urbanski, M., Press, J. B., Dubinsky, B. & Moore, J. B. Jr (1988). *J. Med. Chem.* **31**, 2221–2227. 10.1021/jm00119a026 3184128

[bb9] Sheldrick, G. M. (2008). *Acta Cryst.* A**64**, 112–122. 10.1107/S0108767307043930 18156677

[bb10] Spek, A. L. (2009). *Acta Cryst.* D**65**, 148–155. 10.1107/S090744490804362X PMC2631630 19171970

